# Isoform-resolved transcriptomics reveals stage-specific isoform switching and protein domain remodeling in *Helicoverpa armigera*

**DOI:** 10.1080/21541264.2026.2708591

**Published:** 2026-07-27

**Authors:** Himalshi K. Wickramage, Jack W. Royle, Pawel Sadowski, Kevin J. Dudley

**Affiliations:** a School of Biology and Environmental Science, Queensland University of Technology, Brisbane, Queensland, Australia; b Department of Plant Sciences, University of Saskatchewan, Saskatoon, SK, Canada; c Central Analytical Research Facility, Queensland University of Technology, Brisbane, Queensland, Australia

**Keywords:** *Helicoverpa armigera*, isoform switching, alternative splicing, transcriptomics

## Abstract

Insects exhibit pronounced developmental plasticity, driven by complex regulation of gene expression, including isoform-level modulation across developmental stages. However, the role of isoform-level regulation in the developmental transitions of *Helicoverpa armigera* remains underexplored. Here, we performed transcriptome analysis to characterize gene isoform–specific expression changes between the pupa and adult stages of *H. armigera*. Differential gene-isoform expression analysis revealed that many multi-isoform genes displayed distinct stage-biased regulation that was undetectable at the gene level, uncovering hidden layers of transcriptional complexity. Functional enrichment analyses of differentially expressed multi-isoform genes highlighted MAPK, Hippo, Wnt and apoptosis pathways, consistent with major cellular remodeling processes during the pupa to adult transition. Isoform switching analysis further identified isoform usage shifts between stages, particularly among genes associated with signaling and metabolic regulation. Protein domain and structural modeling revealed that several isoform pairs differed in domain composition, sequence identity, and predicted fold conformation, indicating potential functional divergence in isoform switched genes. Together, these findings suggest that isoform-level modulation may contribute to stage-specific diversification in *H. armigera*. This work establishes a foundation for understanding isoform‑driven developmental plasticity in insects and underscores the importance of isoform-level resolution for elucidating mechanisms of developmental gene regulation.

## 1. Introduction

Transcriptional and post-transcriptional regulation play a key role in morphological evolution by generating proteomic diversity [1]. Among these, alternative splicing (AS) is one of the most important processes underlying transcriptomic and proteomic diversification. Through AS, exons from the same gene are differentially combined or removed from pre-mRNA transcripts, producing multiple mRNA “isoforms” (gene isoforms) that can encode distinct proteins [2,3]. This capacity to generate diverse protein isoforms from a limited number of genes has greatly facilitated the evolution of functional complexity without requiring a proportional increase in gene numbers [4,5]. Isoforms derived from the same gene can be differentially regulated; sometimes activated or suppressed under specific conditions and may even inhibit the activity of other isoforms of the same gene [6]. For instance, the *Bcl-2-like protein 1* (*BCL2L1*) gene encodes two isoforms with opposite roles in cell death in humans: the longer isoform hinders apoptosis, while the shorter isoform promotes it [6].

In insects like honeybees and wasps, sex determination mechanisms can be influenced by AS events; for instance, the *doublesex* (*dsx*) has been found to have 10 female‑specific isoforms, male-specific 6 isoforms in silkworms, which can be exploited to develop female-specific lethality systems [2,7]. Meanwhile, the *Dscam1* gene in *Drosophila melanogaster* can generate more than 38,000 protein isoforms with different binding affinities that enable neurons to self-recognize [6,8]. Despite these examples, many functionally important isoforms remain uncharacterized [2], and most transcriptomic studies still focus on gene-level expression, masking isoform-specific regulation with potential biological relevance.

Phenotypic plasticity can also be regulated through alternative isoform usage, where the relative abundance of different gene isoforms from a single gene shifts between conditions, a process known as “isoform switching” [9–11]. While isoform switching has been reported in a few insect studies focusing on sex and caste differentiation [12,13], its role in coordinating the molecular and metabolic changes across developmental stages remains poorly understood.

The cotton bollworm, *Helicoverpa armigera*, is a holometabolous lepidopteran and one of the most destructive agricultural pests worldwide. It poses a significant threat to more than 181 plants including cotton, rice, maize, tomato, and chickpea, causing substantial losses to the agricultural economy [14,15]. This pest has evolved resistance to chemical insecticides and to several *Bacillus thuringiensis* (Bt) toxins, including Cry1Ac [16], Cry2Ab [17], and Vip3Aa [18], with genomic studies identifying underlying resistance loci and candidate genes in *H. armigera*. These resistance challenges have prompted increasing interest in alternative molecular control strategies such as RNA interference (RNAi) [19]. Although the present study does not directly evaluate pest control applications, isoform-resolved analysis of developmental transitions may help identify candidate molecular targets for future stage-specific control strategies.

Holometabolous insects undergo metamorphosis through the larval-pupal-adult transition, a process controlled primarily by 20-hydroxyecdysone (20E) and juvenile hormone (JH) [20,21]. Metamorphosis involves extensive reorganization of larval tissues into adult structures through tissue histolysis, apoptosis, remodeling, proliferation and differentiation of imaginal primordia and discs [20–22]. This transformation is coordinated by an ecdysone-responsive regulatory cascade, where 20-hydroxyecdysone (20E) activates the Ecdysone receptor/Ultraspiracle protein (EcR/USP) complex, leading to induction of downstream regulators including *Ecdysone-induced protein 74* (*E74*), *Ecdysone-induced protein 75* (*E75*), *Broad-Complex* (*Br-C*), and *Ecdysone-induced protein 93* (*E93*) that drive metamorphosis [21]. Previous studies have conducted gene-level analyses on *H. armigera* metamorphosis from larva to pupa stages [23], but such analyses may overlook isoform-level complexity that shapes stage-specific physiology. This underscores the need to investigate isoform dynamics underlying pupa to adult transition in this pest.

Here, we present the first genome-wide isoform-resolved transcriptomic analysis of pupa to adult developmental transition in *H. armigera*. By integrating differential gene isoform expression, isoform switching, and predictions of protein domain/structural consequences, we reveal that isoform level regulation is associated with stage-specific metabolic and signaling changes. Our study shows that isoform-level resolution reveals expression and isoform-usage patterns that are not captured by gene-level analyses and provides a foundation for exploring isoform-specific molecular targets for future pest management strategies.

## 2. Materials and methods

### 2.1. H. armigera colony rearing

A laboratory colony of *Helicoverpa armigera* was established from eggs supplied by the AgBiTech pesticide company (Toowoomba, Australia) and maintained under controlled environmental conditions (27°C ±1°C, 60% humidity, 16 h light / 8 h dark). Eggs were surface sterilized in 0.25% bleach, rinsed, and air-dried. Larvae were reared individually on an artificial diet until pupation, and emerging adults were provided with a 10% honey solution as a carbohydrate source. Colony maintenance followed the procedure described in Royle et al. [24].

### 2.2. RNA extraction and sequencing

Approximately 100 mg of frozen tissue from each individual was used for RNA extraction from late pupae and newly emerged adults (*n* = 3 biological replicates per stage). Late pupae were staged based on external morphology and collected when the pupal cuticle had turned dark brown, consistent with a stage about 5 days after pupation, and when the eyes had become noticeably dark, indicating proximity to adult emergence [25]. Adults were collected within 24 h of adult emergence. Frozen tissue was ground in liquid nitrogen in an RNase-free mortar and pestle, and the resulting powder was transferred into lysis buffer containing 2-mercaptoethanol. The lysate was homogenized using a spin-column homogenizer and centrifuged at 12,000 × g for 2 min. RNA extraction proceeded using the PureLink™ RNA Mini Kit (Thermo Fisher Scientific) according to the manufacturer’s protocol. RNA concentration and purity were measured using both a NanoDrop spectrophotometer and a Qubit fluorometer. RNA-Seq libraries were prepared using the Illumina TruSeq Stranded mRNA Library Preparation Kit and sequenced as 150 bp paired-end reads on an Illumina NextSeq 500 platform.

### 2.3. Read alignment and quantification

The quality of raw reads was assessed before the alignment step using FastQC software [26]. Then raw reads were mapped to the reference genome of *H. armigera* ASM3070526v1 using the STAR 2 pass mapping method (v2.7.11b). After the first STAR pass [27], splice junctions (SJ.out.tab) were filtered to retain only high-confidence sites by removing those with ≤2 supporting reads, non-canonical junctions, and already annotated junctions. This reduced false positives and redundancy before the second pass [28]. Transcript abundance was quantified using RSEM (v1.3.3) [29]. STAR aligned BAM files were used as input, and quantification was performed against the prepared reference transcriptome using the – paired-end and – strandedness reverse parameters to match the stranded TruSeq library design.

### 2.4. Differential gene isoform expression and alternative splicing

To analyze differential gene isoform expression between adult and pupa stages, RSEM data matrix was used in EBSeq v2.2.0 [30]. Differentially expressed gene isoforms (DEGIs) were identified using a Posterior Probability of Differential Expression (PPDE) greater than 0.95 (equivalent to FDR <0.05), combined with an absolute log_2_fold change (|log_2_FC|) ≥1. The DEGI list was further filtered to retain only genes annotated with more than one mRNA in the reference genome (multi-isoform genes), and this set of multi-DEGIs was used for downstream analyses. Alternative splicing (AS) events in pupa and adult stages were identified using rMATS-turbo v4.3.0 [31]. Five AS event types (SE, A5SS, A3SS, RI, MXE) were quantified, and Percent Spliced In (PSI) values were calculated from the junction-count (JC) output files. Only events with PSI between 0.05 and 0.95 were retained. The distribution of AS events among multi-DEGI genes was visualized using an UpSet plot.

### 2.5. Differential gene expression

Gene-level counts were generated using FeatureCounts via the Rsubread (v2.18.0) package [32]. Then, differential gene expression (DEG) analysis between adult and pupa was conducted employing DESeq2 v1.44.0 [33]. DEGs were defined as having an adjusted *p*-value ≤0.05 and |log_2_FC| ≥1.

### 2.6. Functional enrichment analysis

Gene Ontology (GO) enrichment analysis was conducted using the g:GOSt tool from the gprofiler2 v0.2.3 tool (FDR <0.05) [34]. KEGG pathway enrichment analysis of DEGs was performed using clusterProfiler v4.12.6 [35] with the enrichKEGG function (FDR <0.05). All visualizations were generated in R using the ggplot2 package [36].

### 2.7. Identification of isoform switching genes in developmental stages

Isoform switch analysis was performed with the IsoformSwitchAnalyzeR package in R [11] to detect differential isoform usage (DIU) between adult and pupal stages. Only genes expressing multiple isoforms and with a mean expression above 3 TPM across samples were retained. Individual isoforms were required to have expression above 1 TPM in at least one condition. DIU was quantified as the change in isoform fraction (dIF), defined as the ratio of an isoform’s expression to that of its parent gene. Significant DIUs were identified using the isoformSwitchTestDEXSeq function [37], applying thresholds of |dIF| >0.1 and FDR <0.05. Isoforms were assessed for coding potential with CPC2 [38], and protein domain assignment with PFam [39]. The IsoformSwitchAnalyzeR functions analyzeCPAT() and analyzePFAM() were used to integrate coding potential prediction and domain annotation results.

### 2.8. Protein sequence and structural divergence

The protein sequences belonging to genes that exhibited isoform switching were extracted from the NCBI reference genome to perform sequence alignment. Global pairwise sequence alignment was performed among all protein isoform combinations within each switched gene. Clustal Omega [40] was used for genes with ≥3 isoforms, and EMBOSS Needle [41] was used for pairwise alignment when only two isoforms were present. For each switched gene, the isoform pair with the lowest percentage identity (PID) was selected to represent the highest sequence divergence, and PID distributions were visualized as a histogram. The most sequence-divergent isoform pair from each switching gene was used for structural prediction using ColabFold (AlphaFold2 implementation) [42]. The structures with predicted local distance difference test (pLDDT) >70% were only retained as confidence structures, which were used for structure alignment of protein isoforms for each gene. Then protein structure alignment between protein pairs was performed using the USalign tool [43], and the minimum template-matching score (TM-scores) for each pair was retrieved and plotted in a histogram.

## 3. Results

### 3.1. Gene isoform level differential expression reveals added depth compared to gene-level

On average, 80% of reads were mapped uniquely to the genome, with comparable mapping rates between pupal and adult samples. To capture regulatory complexity, we performed differential expressions at both the gene level (DEG) and gene isoform level (DEGI) using DESeq2 and EBSeq tool, respectively. EBSeq identified 6,229 DEGIs between adults and pupae (FDR <0.05) derived from 4,963 genes (Supplementary Table 1). Among these, 3,532 isoforms were upregulated in adults compared to pupae, while 2,697 isoforms were downregulated.

**Figure 1.**
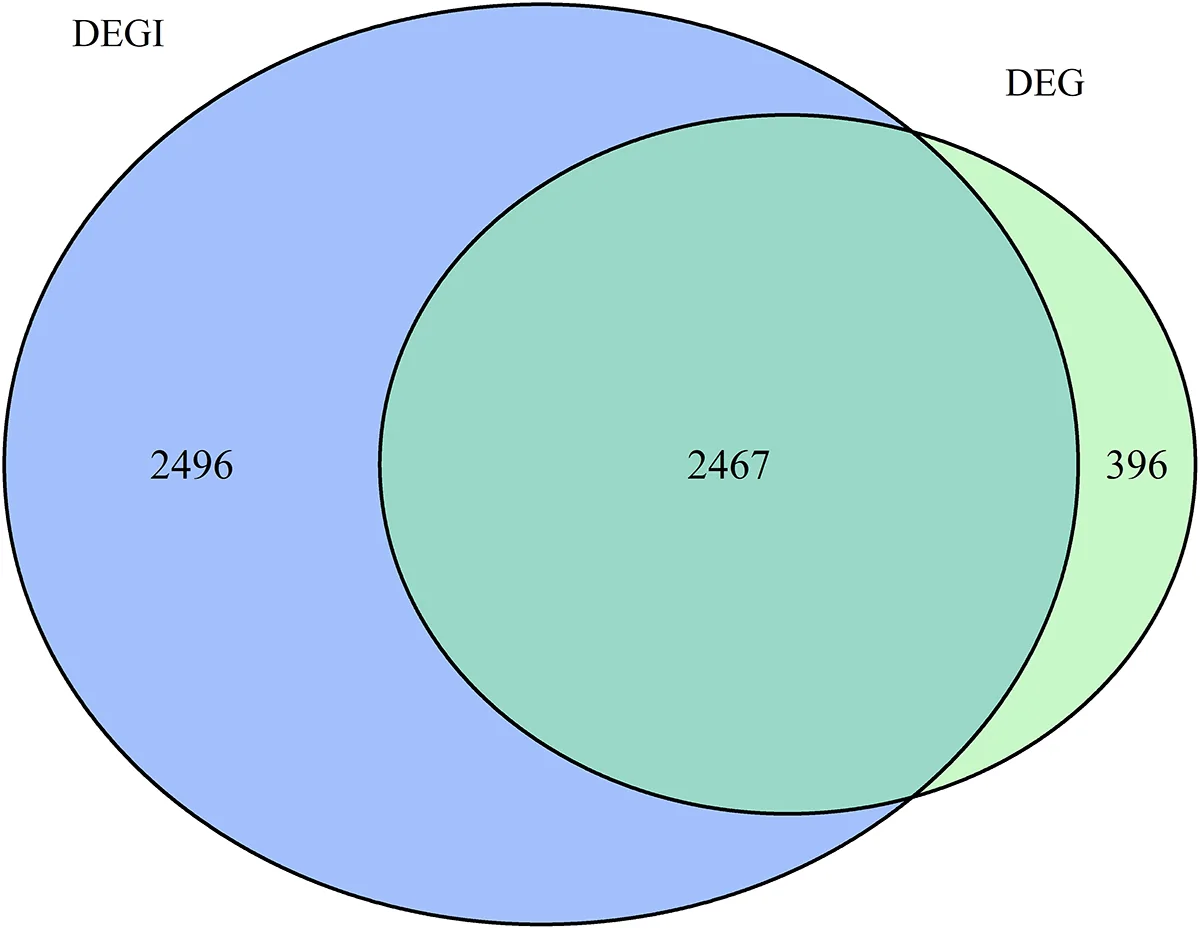
Venn diagram showing the overlap between differentially expressed gene isoforms (DEGIs) and differentially expressed genes (DEGs).

DESeq2 detected 2863 DEGs (FDR  <0.05), of which 1766 genes were upregulated in adults and 1097 downregulated (Supplementary Table 2). Notably, 86% of DEGs overlapped with genes detected in the DEGI analysis (Figure 1). Importantly, DEGI analysis exclusively revealed an additional 2,496 significant genes that were not detected in the DEG analysis, highlighting the added depth of isoform level expression analysis. Several biologically relevant genes, including *Ecdysone-induced protein 75B* (*Eip75B*), *Cyclic-AMP response element binding protein B* (*Crebb*), *Heat shock factor*, and *Insulin-like receptor*, were exclusively detected at the isoform level, demonstrating that isoform-level approaches can uncover regulatory signals masked at the gene level.

### 3.2. Regulation and diversification of multi-isoform genes during metamorphosis

**Figure 2.**
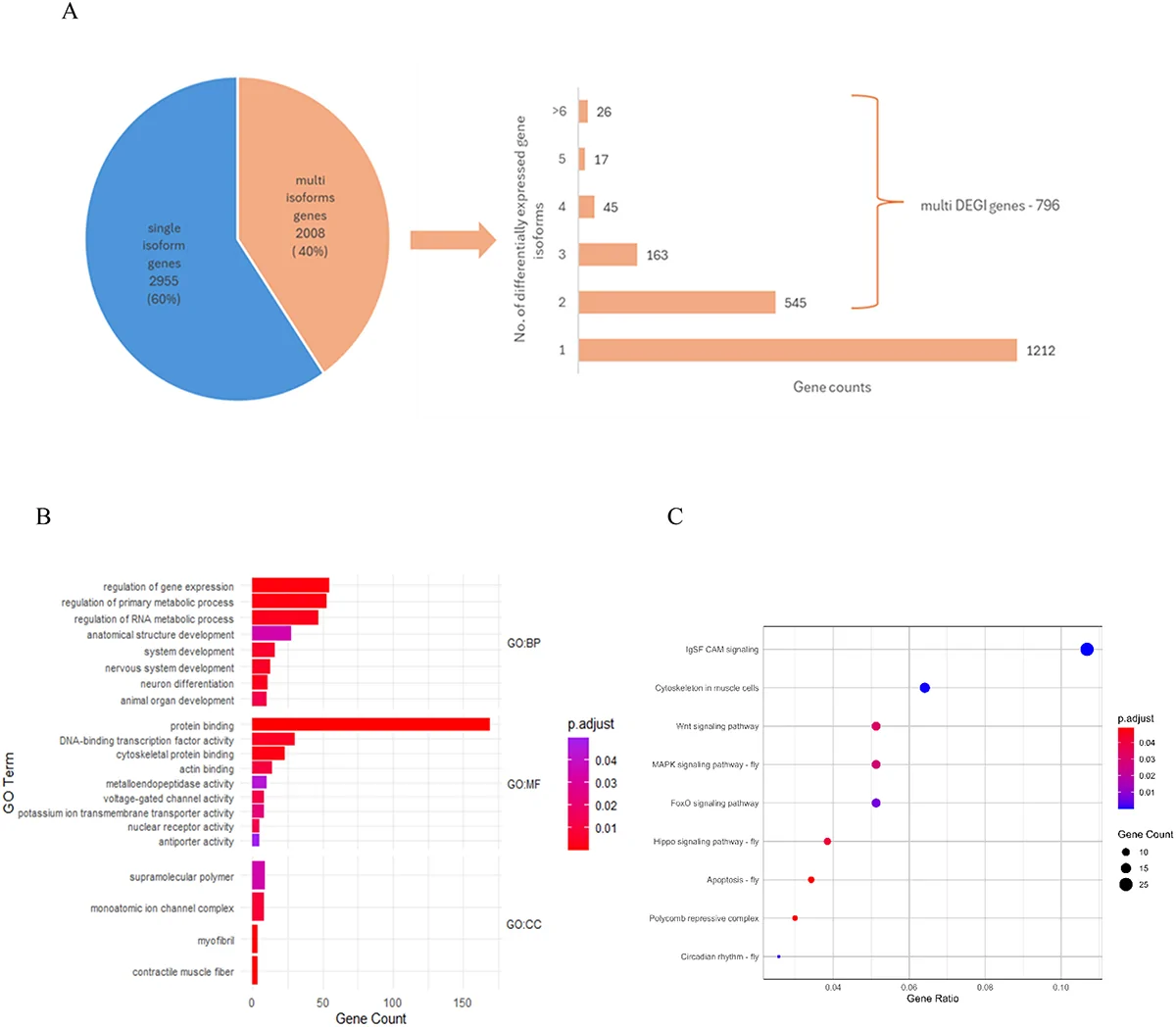
(A) Isoform-level filtering strategy for differentially expressed gene isoform (DEGI) genes. The pie chart shows the proportion of DEGI genes annotated as single isoform versus multi-isoform in the reference genome. The bar chart represents the distribution of DEGIs per multi-isoform gene (B) Bar plot illustrates the enrichment of GO terms associated with multi-DEGI genes. Functional terms were grouped and color coded into three categories: biological process (BP), molecular function (MF), and cellular components (CC). (C) Dot plot illustrates the enriched KEGG pathways among genes with multi-DEGIs. Dot size represents the number of genes associated with each pathway, while dot color indicates the adjusted *p*-value significance level.

To assess isoform‑specific regulation, we focused on genes annotated with multiple isoforms in the reference GTF. Out of 4,963 DEGI genes, 2,008 (40%) were multi-isoform genes, while 2,955 (60%) were single-isoform genes (Figure 2A). Of the multi-isoform genes, 67% were exclusively identified in the DEGI analysis and not detected at the gene level (DEG). Among the multi-isoform genes, 796 contained more than one differentially expressed isoform (multi-DEGI genes). These included 545 genes with two DEGIs, 163 genes with three DEGIs, and up to 1 gene with eighteen DEGIs (Supplementary Table 3). The remaining 1,212 multi-isoform genes had only a single isoform differentially expressed, despite being annotated as multi-isoform in the reference genome. This filtering strategy allowed us to enrich genes where differential regulation occurs across multiple isoforms, providing greater resolution into isoform-level dynamics during developmental stage transition.

GO enrichment and KEGG pathway enrichment were performed to explore the functional importance of these multi-DEGI genes and visualized in a Bar plot (Figure 2B) and Dot plot (Figure 2C), respectively. GO terms including cytoskeletal protein binding, metalloendopeptidase activity, animal organ development, system development, anatomical structure development, neuron differentiation, regulation of metabolic process, and myofibril were enriched among multi-DEGI genes. These enriched terms suggest that multi-DEGI genes are associated with developmental processes, cellular reorganization, and metabolic regulation during metamorphosis. The KEGG pathways also enriched in Hippo signaling pathway, Apoptosis, Wnt signaling pathway, MAPK signaling pathway, FOXO signaling pathway. Notably, some of the multi-DEGI genes included *Eip75B*, *E74*, *Br-C*, and *EcR*, which are key components of the ecdysone signaling pathway and are well known to regulate developmental transitions during insect metamorphosis [21,23]. Together, these enrichment results suggest that multi-DEGI genes are associated with pathways and functional categories related to programmed cell death, growth control, and developmental regulation during metamorphosis.

**Figure 3.**
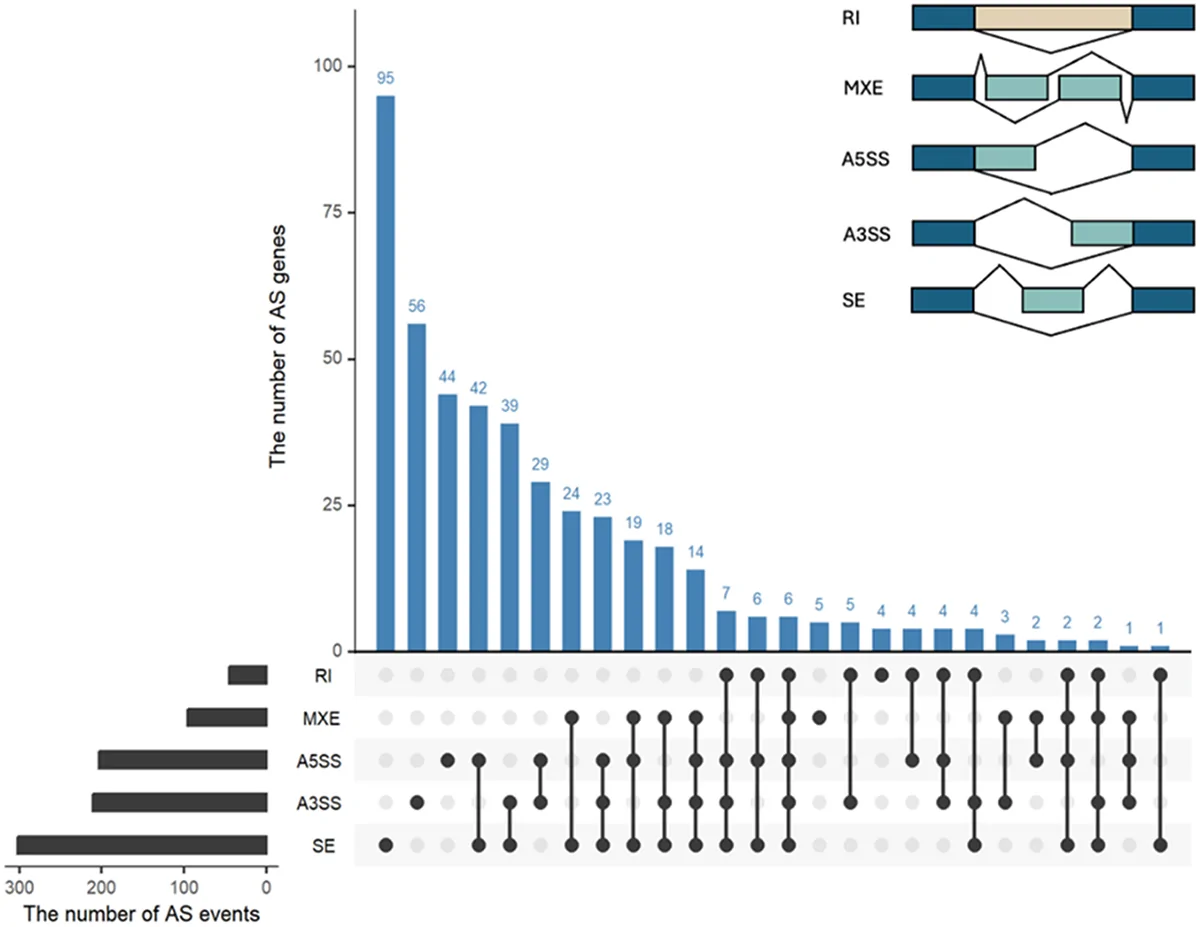
Upset plot shows the distribution of alternative splicing (AS) events in multi-DEGI genes. Horizontal bars (left) indicate the total number of events per AS category: SE, MXE, RI, A3SS, and A5SS. Vertical bars (top) represent the number of genes undergoing one or more AS event combinations.

To investigate the alternative splicing (AS) events in these multi-DEGI genes, rMATs tool was used, and Upset plot was generated to visualize the genes with multiple types of AS events (Figure 3). We identified AS events in 459 genes out of 796 DEGI genes (Supplementary Table 4), covering five types of events: skipped exon (SE), mutually exclusive exon (MXE), retained intron (RI), alternative 3’ splice site (A3SS), and alternative 5’ splice site (A5SS). The highest proportion of multi-DEGI genes exhibited SE events, followed by A3SS and A5SS. A total of 204 genes (44.4%) contained a single AS event, while the remaining 255 genes (55.6%) displayed two or more splicing patterns, forming 21 combinations of complex AS types. Among the 6 genes displaying all splicing patterns, Myosin heavy chain (Mhc) was notable for having the highest number of adult-upregulated isoforms (18), indicating that alternative splicing significantly contributes to gene regulation in stage-specific transition.

**Figure 4.**
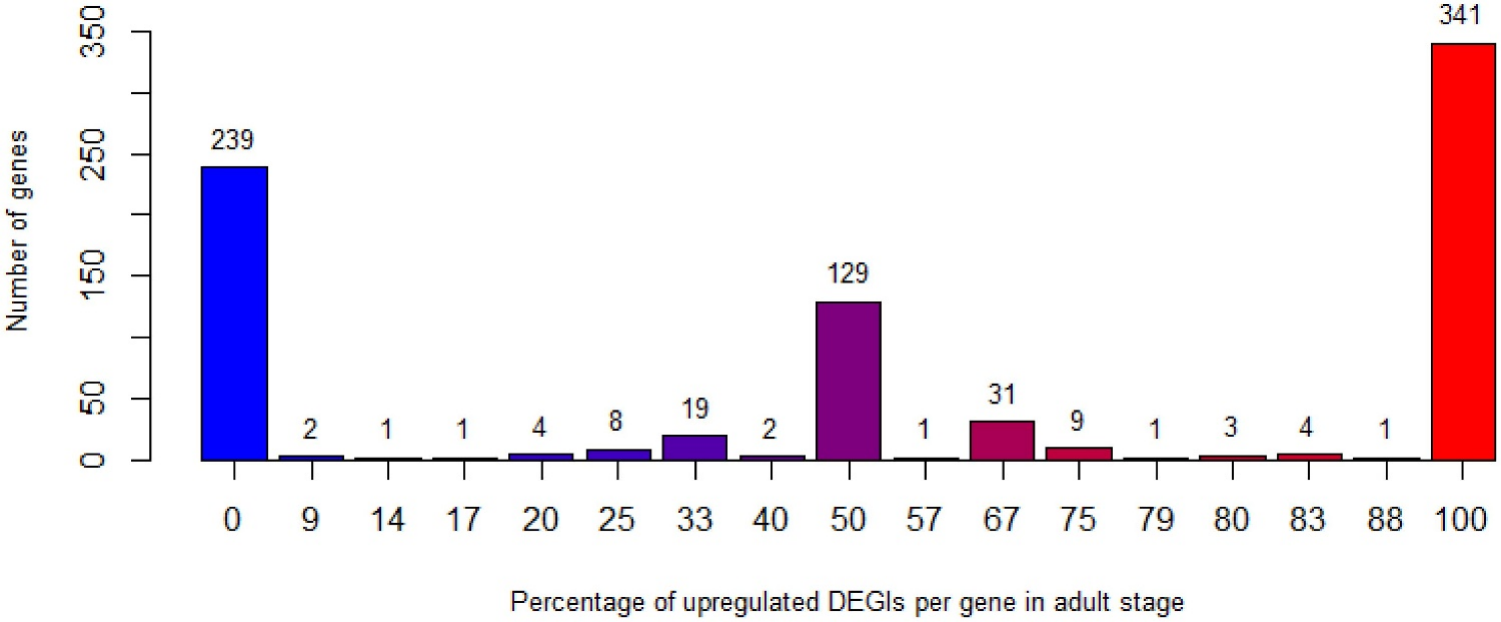
Isoform-level expression bias in multi-DEGI genes between pupal and adult stages of H. armigera. Bars show the distribution of genes based on the percentage of isoforms upregulated in adults (0% = all isoforms upregulated in pupae; 100% = all isoforms upregulated in adults). The color gradient indicates stage bias from blue (pupa-biased) to red (adult-biased), with intermediate shades representing mixed regulation.

We then quantified the proportion of upregulated isoforms per gene in adults compared to pupae to further characterize expression patterns of multi-DEGI genes (Figure 4). This analysis categorized genes into 3 main types: the genes with all isoforms upregulated in adults (100%), those with all isoforms downregulated (0%) in adults (upregulated in pupae), and genes with mix of upregulated and downregulated isoforms in adults (intermediate values between 0% and 100%). Notably, 129 genes showed a balanced pattern with 50% of isoforms upregulated in each stage. Genes closer to 100% exhibited a greater bias toward adult-upregulated isoforms, whereas those closer to 0% were predominantly pupa-upregulated. In total, 216 genes displayed mixed regulation, of which 20 were detected only at the isoform level and were not identified in gene-level differential expression analysis. This indicates that gene-level approaches can mask biologically relevant events that are only revealed through isoform-level analysis. Importantly, several key regulators of development transition, including *MAP4K3-like protein hppy* (*Hppy*), *Ei75B*, *Muscle-specific protein 300k Da* (*Msp300*), *Crebb*, *Transcriptional coactivator yki* (*Yki*), and *Death-associated protein kinase‑related* (*Drak*), were identified exclusively in this mixed-regulation category of DEGI genes. These findings highlight the added biological insights gained from isoform-level analysis and prompted further investigation into isoform usage differences among multi-isoform genes.

### 3.3. Identification of isoform switching events with functional consequences during metamorphosis

To investigate isoform usage differences between conditions in multi-isoform genes, we used IsoformSwitchAnalyzeR, which defines isoform switching events as changes in the functional potential between two isoforms (|dIF| >0.1, FDR <0.05) that can exert significant biological effects [11]. In total, 700 isoform switching events were detected across 905 isoforms belonging to 488 genes (Supplementary Table 5). Of these, 405 (83%) genes overlapped with DEGI analysis, including 108 multi-DEGI genes with mixed regulation, whereas only 77 (16%) genes overlapped with DEG analysis. This indicates that gene-level expression analysis can mask switching events that become evident only at the isoform level.

**Figure 5.**
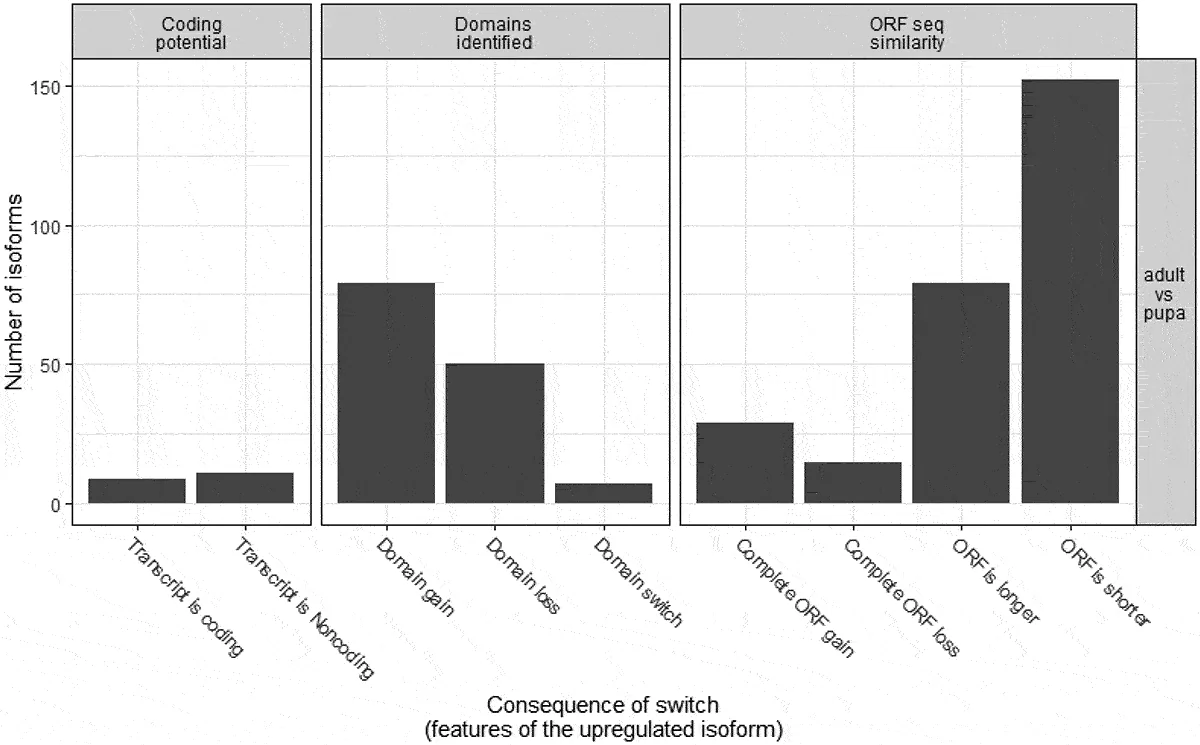
Functional consequences of isoform switching between pupa and adult stages. Bar plots show the number of upregulated isoforms predicted to undergo changes in coding potential, protein domain composition, and ORF sequence during switching events.

IsoformSwitchAnalyzeR further predicted the functional consequences of the upregulated isoforms in pupae within switched genes (Figure 5). Across all switching events, the most frequent changes involved the open reading frame (ORF) length, with 142 genes showing isoform length alterations (Supplementary Table 6). In addition, 64 genes exhibited gain, loss, or switching of protein domains in isoforms (Supplementary Table 7), while 9 genes were predicted to undergo changes in coding potential in isoforms. These results indicate that isoform switching contributes to functional diversification of transcripts, particularly through modifications in ORF structure and protein domains, thereby supporting stage-specific regulation during metamorphosis.

KEGG pathway enrichment of switched genes highlighted terms such as the MAPK signaling pathway, nicotinate and nicotinamide metabolism, cytoskeleton in muscle cells, and circadian rhythm (Supplementary Figure 1A). Consistent with these findings, GO enrichment revealed categories including cytoskeletal protein binding, troponin complex, and myofibril, indicating that many isoform switching events occur in muscle-related genes (Supplementary Figure 1B). In addition, GO terms related to metabolic processes, molecular binding, and vesicle organization were enriched, suggesting that isoform switching contributes to metabolic regulation during metamorphosis.

**Figure 6.**
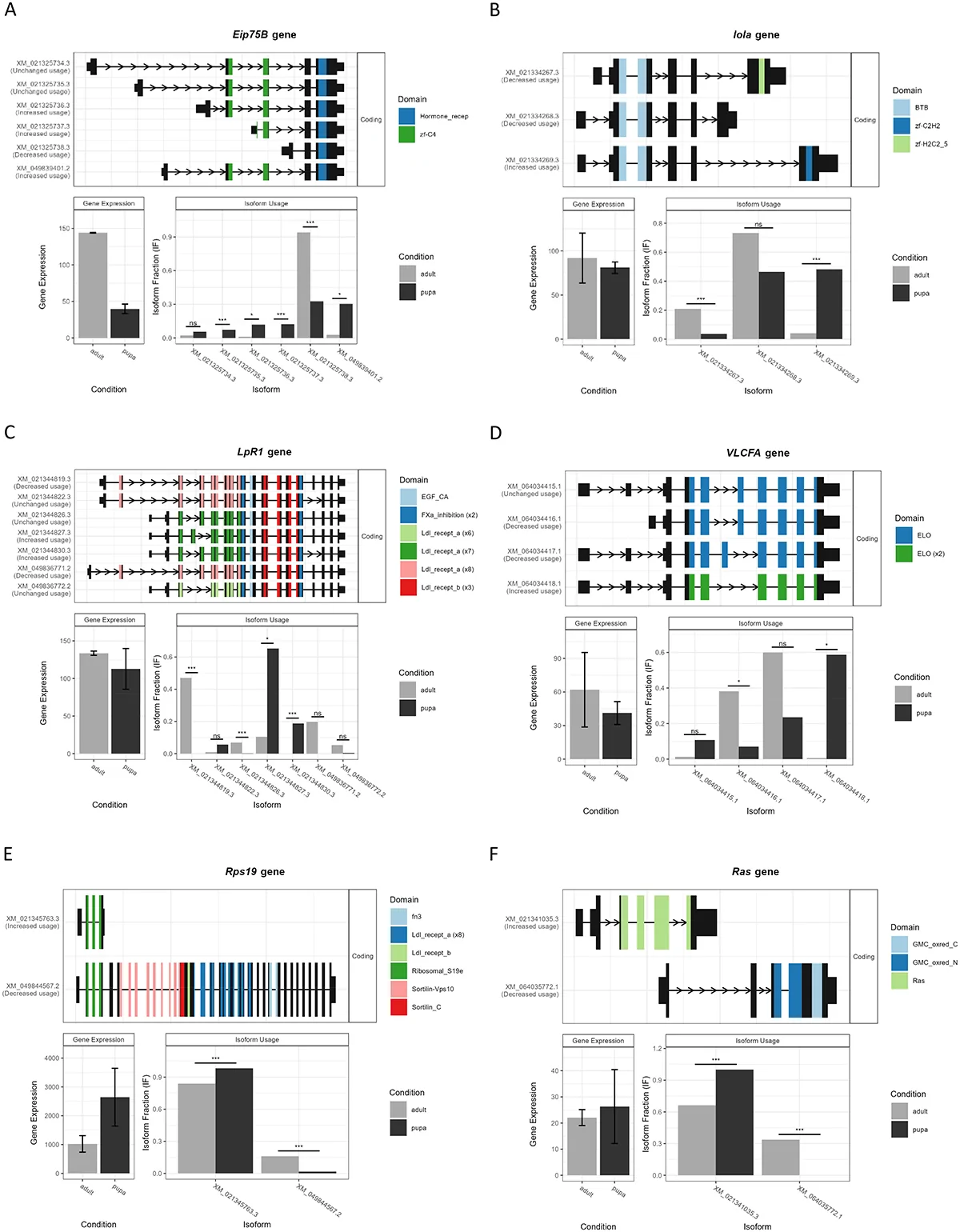
Switch plots of isoform switching in developmentally relevant genes and predicted functional shifts (A)*Eip75B*(*ecdysone-induced protein 75B*) - pupal isoform retains both zfC4 domains; adult isoform lacks one zfC4 (B)*lola*(*longitudinals lacking*) - pupal isoform contains zf_C2H2 motif; adult isoform uses zf_H2C2_5 (C)*LpR1*(*lipophorin receptor 1*) - pupal isoforms have lost aLdl_receptor_a repeat compared to adult isoform (D)*very long chain fatty acid elongase AAEL008004-like*(*VLCFA*) - pupal isoform includes an extra ELO domain relative to adult isoform (E)*RpS19a*- adult SORL1 isoform have multiple domains (sortilin, LDL_receptor, FN3); RpS19a have only ribosomal_s19e domain (F)*Ras*- pupal isoform contains RAS domain; adult isoform includes GMC_oxred domains *—*p*-value <0.05, ***—*p*-value <0.001, ns—no significant difference.

We investigated some isoform switching events in which both protein domain changes and ORF alterations occur between pupa and adult isoforms. Figure 6(A) illustrates isoform switch in the *Eip75* gene between pupa and adult stages. *Eip75* encodes a transcription factor in the ecdysone signaling cascade, where it regulates processes like cellular differentiation and circadian rhythm [12]. In the pupal isoform, both DNA binding zinc finger (zfC4) domains are retained in a longer ORF, whereas the adult upregulated isoform lacks one zfC4 domain and is shorter, which is consistent with the *D. melanogaster* E75B isoform that contains only a single zinc finger and is unable to bind DNA directly [45]. This suggests a possible functional shift: the pupa biased isoform may act as an activator in the 20E pathway, while the adult biased isoform acts as heterodimer partner. In *D. melanogaster*, the single zinc finger E75B isoform is known to heterodimerize with hormone receptor 3 (HR3) and participate in 20E‑dependent developmental pathways [45].

Isoform switching events in *longitudinals lacking* (*lola*) gene exhibit a coding domain switch and ORF change in the pupa upregulated isoform when compared to the adult upregulated isoform (Figure 6B). It is a transcription factor involved in regulating adult midgut homeostasis, ovary development, neuron stem cell and male germline maintenance and differentiation, axon growth and guidance [46]. The shorter isoform with a zf_C2H2 domain predominates in pupae, while a longer isoform bearing zf_H2C2_5 prevalent in adults suggests a switch in isoform usage that could underpin stage-specific gene regulatory programs.

Figure 6(C) illustrates an isoform switching event in the *Lipophorin receptor 1*(*LpR1*) gene, which functions primarily in lipid uptake and transport. In this switch, two pupa upregulated isoforms have lost one Ldl_receptor_a repeats relative to the adult upregulated isoform, suggesting that adults may express a variant with enhanced lipid binding capacity. The Ldl_receptor_a repeats are essential for ligand binding in LDL receptor family proteins [44]. Thus, changes in the number of these repeats between life stages likely alter receptor affinity or specificity, potentially adjusting lipid uptake needs between pupa and adult.

Figure 6(D) illustrates an isoform switch in the *very long chain fatty acid elongase AAEL008004-like*(*VLCFA elongase*) gene, where the pupa upregulated isoform contains an additional ELO domain compared to the adult upregulated isoform. In insects, elongases (ELOs) are central to fatty acid chain extension and have been implicated in key physiological roles such as cuticle formation, pheromone biosynthesis, and reproductive function [47]. Given the enzymatic role of ELO proteins in lipid metabolism, the dual-domain pupal isoform may support high demands for lipid remodeling and membrane biogenesis during metamorphosis. In contrast, the adult single domain isoform might tailor to adult lipid metabolic needs such as reproduction.

The *Ribosomal protein S19a* (*RpS19a*) gene has lost protein domains in pupa upregulated isoform of small ribosomal subunit protein eS19A compared to the adult upregulated isoform sortilin-related receptor (Figure 6E). The adult upregulated Sortilin-related receptor (SORL1) protein is 2,351 amino acids long and retains multiple functional domains (sortilin, LDL_receptor, and FN3), whereas RpS19a is only 153 amino acids and lacks those domains. SORL1 protein is involved in intracellular vesicle trafficking and lipid transport and metabolic regulation [48]. Thus, the upregulation of SORL1 in adults may support increased lipid uptake, intracellular transporting [48], and metabolic demands in adult tissues, while ribosomal protein upregulation in pupae suggests maintenance of fundamental translation machinery during that stage.

Similarly, in the *Ras-related protein M-Ras* gene, we detected an isoform switching event (Figure 6F) that suggests divergent functional potential. The pupal upregulated isoform of M-Ras includes a Ras domain gain, whereas the adult prominent isoform is ecdysone oxidase (EO) isoform with GMC_oxred domains. Ras proteins influence ecdysone release and stage-specific remodeling, supporting a role in metamorphic regulation [49,50]. EO is an enzyme known to inactivate ecdysone, thereby modulating ecdysteroid signaling during metamorphosis [51,52]. The occurrence of such divergent predicted protein products from the same gene suggests that isoform switching may broaden stage-specific functional potential during metamorphosis. These observations led us to further examine whether isoform switching generally results in protein-level divergence across genes.

### 3.4. Isoform switching can drive protein divergence

**Figure 7.**
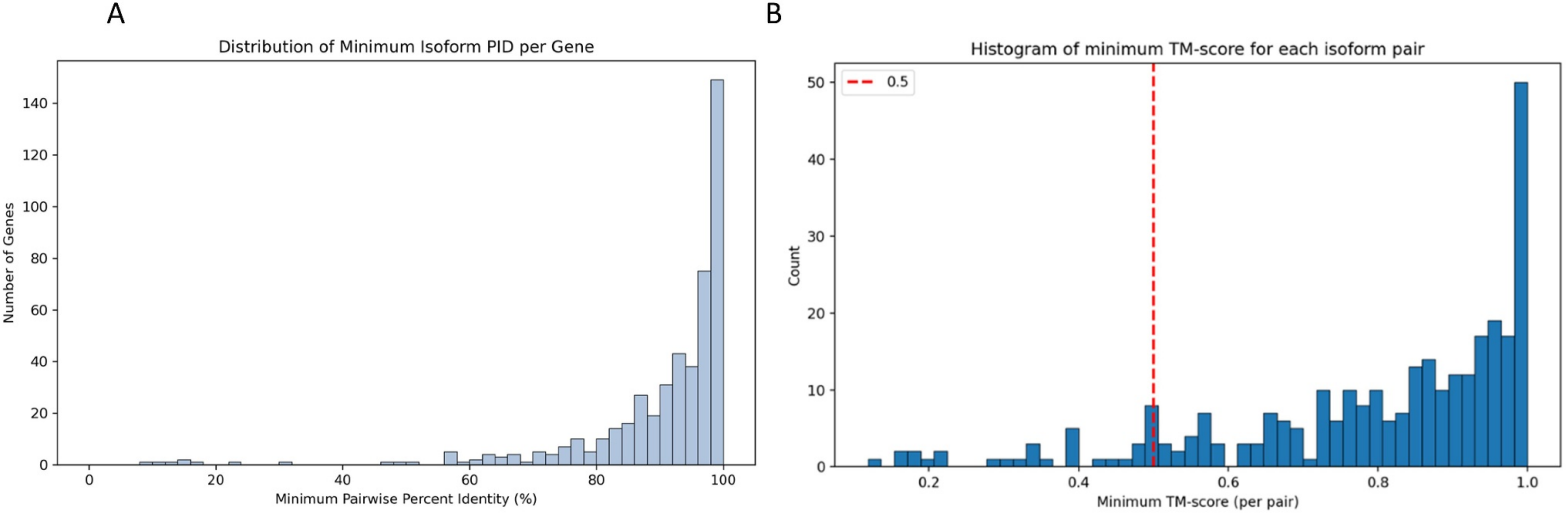
(A) Distribution of sequence similarity between isoform pairs in switched genes. Histogram showing the minimum pairwise percent identity (PID) between protein-coding isoforms of genes undergoing isoform switching (B) structural divergence among isoform pairs based on TM-score. Histogram showing the distribution of minimum TM-scores across isoform pairs from isoform-switched genes. The red dashed line marks the 0.5 threshold.

We assessed both protein sequence divergence and structural divergence among isoforms in the switching genes to evaluate whether isoform switching has functional consequences. For sequence divergence, we computed the minimum PID between all pairs of protein coding isoforms per gene (i.e., the most divergent pair) across genes showing isoform switching (Supplementary Table 8) and visualized in Figure 7(A). While many genes show high PID, 9 genes fall below 40%, a level near the lower bound of the safe zone for structural inference [53], indicating substantial sequence divergence. This suggests that, in these genes, isoform switching produces markedly different amino acid sequences that may drive functional divergence. Examples of sequence divergent cases include Ribosomal protein S19a and the Ras-related protein M-Ras gene, which were highlighted from the previous switch plot analyses.

To further investigate whether sequence divergence corresponded to structural changes, we predicted tertiary structures using ColabFold, retaining only models with confidence pLDDT >70%, which yielded structural models for 299 genes. These structures were aligned using US-align to compute the minimum TM-score per gene (Supplementary Table 9). As proposed by Xu and Zhang [54], a TM-score >0.5 generally indicates that two proteins share the same topology, whereas protein pairs with a TM-score  <0.5 are expected to adopt different topologies. Consistent with this benchmark, the TM-score histogram (Figure 7B) shows that while many isoform pairs retain structural similarity, 31 genes had TM-scores <0.5, indicating fold-level structural divergence. Among the genes exhibiting both low PID and low TM-score, several stand out as particularly striking examples of structural and functional divergence. Among the genes with both low sequence identity and low TM-score, in addition to switched isoforms in *Ras* gene, other notable cases include *Ciao1* gene, whose isoforms switch to NIF3-like variants; *Transducin β-like protein 2*, with one isoform containing a WD40 scaffold domain and another lacking it (exonuclease GOR-like form); *FRA10AC1 homolog*, where isoforms switched to zinc-finger protein; and *tubulin-specific chaperone C*, switching to a CEBPZOS-like protein. These results suggest that some isoform switching genes undergo both sequence and structural divergence, indicating that switching may drive functional shifts during metamorphosis.

## 4. Discussion

This study provides the first genome-wide characterization of isoform-level regulation and switching during metamorphosis in *H. armigera*. While previous transcriptomic analyses of this species, such as [23], profiled brain and fat body during the larva to pupa transition at the gene expression level, our isoform-resolved analysis of the pupa to adult transition revealed an additional layer of developmental regulatory complexity. Some of those regulatory genes and pathways reemerged across our isoform-resolved analyses. In particular, eight *Br-C* isoforms were upregulated in pupae, *Eip75B* showed mixed isoform regulation, with four isoforms upregulated in pupae and one in adults, three *E74* isoforms highly expressed in pupae. Gao et al. [23] likewise reported high expression of *Br-C*, *E75* and *E74* genes at the prepupal stage. Thus, our isoform-level results for this ecdysone signaling genes during the pupa to adult transition are broadly consistent with this earlier gene-level framework.

In comparison, Gao et al. [23] detected elevated *E93* at prepupal stage compared to larva, whereas in our dataset one *E93* isoform was upregulated in adults relative to pupa, consistent with the established role of *E93* in promoting adult morphogenesis [55]. Overall, these patterns are also broadly consistent with the endocrine model summarized by Truman and Riddiford [56], in which *Br-C* specifies the pupal stage and *E93* promotes adult differentiation. Accordingly, the pupal enrichment of *Br-C* isoforms and the reduced abundance of one *E93* isoform in pupae are consistent with a shift from pupal to adult regulatory states across metamorphosis [55,56]. This interpretation is also supported by tissue context studies of Broad expression. In *Manduca sexta*, Broad is induced in the epidermis before the wandering stage, whereas imaginal discs and primordia express Broad earlier as they enter morphogenesis, indicating that *Br-C* is activated in tissues as they become committed to pupal differentiation [56,57]. *E93* has likewise been identified as a conserved “adult specifier” gene in holometabolous insects [55]. Consistent with this broader endocrine context, three *EcR* isoforms were significantly upregulated in pupae. In *D. melanogaster*, the *EcR* gene produces EcR-A, EcR-B1, and EcR-B2, which show distinct developmental expression patterns, with EcR-A predominant in imaginal discs of larval stage for pupal and adult structure development, EcR-B1 enriched in tissues undergoing apoptosis and EcR-B2 predominantly expressed in Malpighian tubules. This suggests that the coordinated upregulation of multiple EcR isoforms in *H. armigera* may likewise reflect tissue or process-specific functions during metamorphosis [21,58].

A similar endocrine pattern has also been reported in *Bombyx mori* wing disc development, where 20E signaling genes including *E74*, *E75* and *HR3* were upregulated during larva to pupa metamorphosis. The same study also reported pupal stage upregulation of the Achaete-scute homolog (*ASH2*), which is required for wing scale formation [59]. This is consistent with our observation that *E74*, *Eip75B*, *HR3* and achaete-scute complex protein T3 showed pupal-biased isoform expression in *H. armigera*. Together with the pupal upregulation of several cuticle‑associated transcripts, these patterns suggest that part of the pupal-biased isoform signal may be associated with wing and cuticle development during metamorphosis, although tissue-specific profiling will be needed to confirm this interpretation.

Juvenile hormone metabolism also showed isoform-level regulation during the pupa to adult transition. In our dataset, eight *juvenile hormone esterase* (*JHE*) transcripts were adult-biased, whereas *LOC110383157* gene showed opposite isoform regulation, with two pupa-biased transcripts and one adult-biased transcript. Since *JHE* hydrolyzes JH and contributes to the regulation of JH titer, the predominance of adult-biased JHE transcripts may reflect increased JH turnover during adult maturation rather than simple suppression of JH activity. This is relevant because *H. armigera* endocrine data show that JH is low during prepupal/pupal stages but becomes active again after adult emergence, with titers peaking in 3-day old adults [60]. Thus, adult-biased JHE transcripts may contribute to regulating renewed adult JH signaling, whereas pupa-biased isoforms may contribute to JH clearance during pupal development.

Although *Kr-h1* is classically associated with JH‑dependent anti-metamorphic signaling, previous studies show that it can also be expressed during adult development in a tissue‑specific manner. In *D. melanogaster*, *Kr-h1* is upregulated during most of adult development in abdominal epidermis [61], whereas in the lepidopteran *B. mori*, *BmKr-h1* is highly expressed in late pupal and adult ovaries and promotes oocyte maturation [62]. Therefore, the adult biased *Kr-h1* signal in our dataset may reflect stage‑ and tissue‑specific functions in adulthood.

Gao et al. [23] also showed that multiple neuropeptide receptor genes in the *H. armigera* brain were highly expressed during the wandering stage of larval development. Among related transcripts in our DEGI list, octopamine and 5-hydroxytryptamine associated transcripts were reduced in pupae relative to adults, suggesting that these signaling pathways may remain developmentally regulated during the later transition to adulthood. Insect studies have shown that octopamine signaling contributes to neural and behavioral processes such as appetitive memory recall [63].

These endocrine and neuropeptide associated expression patterns were accompanied by isoform-level changes in genes previously implicated in remodeling-related processes, including extracellular matrix degradation, proteolysis, and fat body remodeling [64]. In *H. armigera*, fat body dissociation during pupal development has been functionally associated with matrix metalloproteinase activity, extracellular matrix (ECM) remodeling, and transcriptional activation of lipid metabolism pathways that provide energetic support for pupal development [64]. Consistent with this model, our results showed two matrix metalloproteinase 2 (*MMP2*) isoforms upregulated in pupal samples, which also agrees with the prepupal upregulation of *MMP2* reported by Gao et al. [23], suggesting that *MMP2* associated ECM remodeling may be active during the pupal phase of the pupa to adult transition. A recent study also showed that 20E promotes brain development during metamorphosis by *MMP2* upregulation, thereby supporting neural cell proliferation and nutrient transporter expression [65]. Gao et al. [23] further implicated cathepsins, caspases genes in larva to pupa tissue remodeling, particularly in the fat body. In contrast, our pupa to adult comparison showed adult biased expression of *Caspase 1*, *Cathepsin L*, *Cathepsin B-like* and two *Cathepsin K* transcripts, suggesting that proteolytic and programmed cell death pathways may remain active during the adult stage. This adult-biased pattern is consistent with previous work showing that *H. armigera* Cathepsin B-like proteinase (HCB) is activated during the pupa to adult metamorphosis and is involved in adult fat body decomposition during aging and oogenesis [66]. Caspases are major effectors of apoptosis in Lepidoptera and mediate programmed cell death during metamorphic tissue remodeling [22]. Together, these observations indicate that the apoptosis and proteolysis-related signals identified in our dataset are consistent with known developmental remodeling processes in *H. armigera*, although direct functional validation of the individual isoforms identified here remains necessary.

In addition to cathepsin‑related proteolysis, we also observed stage-biased regulation of several serine protease inhibitor (SPI) transcripts, including serine protease inhibitor 77 Ba, serine protease inhibitor 3, serine protease dipetalogastin inhibitors. Most of these transcripts were more highly expressed in adults than in pupae, although two transcripts showed the opposite pattern. This is broadly consistent with *Spodoptera frugiperda*, where SPI family genes showed predominantly adult-biased but overall heterogeneous developmental expression, suggesting roles in immune regulation and metamorphosis [67].

Many developmental-related multi-DEGI genes, including *Eip75B*, *Crebb*, Yki, *MMP2*, and *Msp300,* showed isoform-specific regulation undetectable at the gene level, underscoring how collapsing isoform data can mask functionally important developmental signals.

Functional enrichment of multi-DEGI genes highlighted pathways central to metamorphosis, including MAPK, Hippo, and Apoptosis. These pathways are associated with cell proliferation, tissue regeneration and developmental remodeling in insects. Interestingly, they overlap with pathways enriched in differentially methylated regions identified between pupae and adults of *H. armigera* [24]. It suggests that DNA methylation may have a potential role in isoform regulation during development, which requires further study.

Furthermore, our analysis revealed that approximately 58% of AS occurred within multi-DEGI genes, underscoring the contribution of post-transcriptional regulation to stage-specific phenotypic differentiation. The most prevalent type of AS is SE, which aligns with other higher eukaryotic species except plants and fungi [1].

Among the multi-DEGI genes, 239 genes contained isoforms that were specifically upregulated in pupae, and 341 genes contained isoforms specifically upregulated in adults, indicating that stage-specific isoform usage is widespread and may reflect functional specialization, although this requires further experimental validation. In addition, several multi-DEGI genes showed mixed regulation, where different isoforms of the same gene were upregulated in different stages. This pattern indicates that individual isoforms are expressed selectively depending on developmental context, underscoring the importance of studying isoform usage rather than gene-level expression alone.

This pattern was also evident among digestion‑related multi-DEGI genes. Although the first genome-wide study of *H. armigera* by Pearce et al. [68] did not investigate isoform-level regulation during the pupa to adult transition, it showed that most trypsin and chymotrypsin family members are associated with larval digestive tissues, particularly midgut. In our dataset, multiple isoforms of trypsin-7 (three isoforms), trypsin-1 (two isoforms), antichymotrypsin-2 (two isoforms), and trypsin-5-like (two isoforms) were upregulated in adults, whereas trypsin 3A1 showed mixed isoform regulation, with one isoform upregulated in pupae and another in adults. Although our data are not tissue resolved, these annotations provide a plausible digestive tissue context for some of the stage-biased isoforms identified here.

The discovery of stage-specific isoform switching in key developmental and metabolic genes, such as *Eip75B*, *Lola*, *LpR1*, and *M-Ras*, identifies candidate molecular transitions associated with pupa and adult physiology. Importantly, some of these switched isoforms differ in coding sequence, domain composition, or predicted structural conformation, indicating that isoform switching can remodel protein structure and function rather than simply alter transcript abundance. In *D. melanogaster*, *Eip75* isoforms have been shown to perform opposing functions in female reproduction, with *E75A* promoting apoptosis in the egg chamber and *E75B* suppressing it [69]. The pupa to adult isoform switching we observe in *H. armigera* likely reflects a metamorphic reprogramming event rather than sex-specific regulation, suggesting that *Eip75* isoform functions may be conserved across insects. Likewise, the *lola* gene represents a large transcription factor family with at least 19 isoforms in *Drosophila*, each exhibiting distinct DNA binding affinities and neural wiring functions [1]; the stage-biased isoform usage we detected in *H. armigera* may similarly underlie developmental regulation of neurogenesis and tissue remodeling. The *LpR1* switch may indicate stage-specific changes in lipid transport during the pupa to adult transition, consistent with previous studies showing tissue-specific expression of insect lipophorin receptor splice variants in lipid handling tissues such as the fat body and ovary [44]. The adult-specific expression of the *M-Ras* isoform corresponding to *ecdysone oxidase* (*EO*) suggests metabolic adaptation of ecdysteroid signaling during adulthood. In *B. mori*, *EO* is expressed in mature ovaries, where it contributes to maternal provisioning of ecdysteroid precursors [52].

Moreover, the identification of Tropomyosin, Troponin T skeletal muscle, Myosin heavy chain and Muscle LIM protein 1, together with enrichment of isoform switched genes for myofibril, contractile muscle fiber, troponin complex, and striated muscle thin filament GO terms, suggests that some isoform switching events may be associated with muscle development and functional maturation during metamorphosis. This interpretation is consistent with previous work in Drosophila showing that alternative splicing of a muscle tropomyosin gene generates tissue-specific isoforms expressed during embryonic myogenesis and adult thoracic flight muscle development [70]. Similarly, Troponin T isoforms are regulated by alternative splicing in a muscle and developmental stage-specific manner, including in Drosophila flight muscle [71]. These findings suggest that isoform switching in contractile genes in *H. armigera* may reflect stage-specific regulation of muscle associated transcripts during the pupa to adult transition. However, the functional significance of these individual isoform changes in *H. armigera* remains to be determined experimentally.

Isoform switching was also detected for integrin subunit alpha inflated (*if*), an orthologue of the Drosophila αPS2 integrin subunit. Integrins are central mediators of cell-extracellular matrix adhesion, and studies in Drosophila have shown that integrin‑mediated cell-ECM interactions regulate epithelial cell shape changes during wing morphogenesis [72].

Protein sequence identity and structural similarity were high in the majority of isoform switching genes, indicating most alternative isoforms preserve core fold architecture. However, a subset of isoform pairs displayed structural divergence despite moderate sequence identity, consistent with observations by Song et al. [73] that some alternatively spliced isoforms can adopt distinct folds even at relatively high sequence similarity. This suggests that while isoform switching typically conserves sequence integrity, specific events can remodel protein topology, potentially leading to functional diversification. Moreover, some isoform switching genes showed divergence at both the sequence and structural levels, including *M-Ras*, *Ciao1*, *Transducin β-like protein 2*, *FRA10AC1 homolog*, and *Tubulin-specific chaperone C*, suggesting that isoform switching can generate structurally distinct protein variants with the potential for functional specialization. In several of these structurally divergent cases, isoform switching also coincided with changes in domain composition (e.g., WD40, zinc finger, NIF3-like, Ras) and repeat organization. These structural variations align with mechanisms reported by [73] that alternative splicing can destabilize protein conformations, modify transmembrane domains, and generate variability in repeat regions. Such alterations likely contribute to isoform-specific functional specialization in *H. armigera*.

Despite providing new insights into isoform-level regulation during developmental transitions, this study has a few limitations. The use of short-read RNA sequencing, while effective for quantifying expression, may not fully resolve complex splice variants or capture lowly expressed isoforms. Future studies integrating long-read sequencing technologies (e.g., Iso-Seq or Oxford Nanopore) together with methylome profiling will be valuable for achieving a more complete view of isoform diversity and its regulation in *H. armigera*. Validating stage-specific isoforms using proteomics will reveal which switching events are functionally meaningful. Given that this study used whole body samples, the tissue origin of the observed isoform-level signals remains unresolved. Future tissue-specific transcriptomic profiling will be needed to determine the tissue specificity of candidate isoform switches. Together, these future approaches will deepen mechanistic understanding of isoform diversity and may enable identification of isoform-specific vulnerabilities for stage-targeted pest management strategies.

## 5. Conclusion

Isoform-resolved transcriptomics revealed regulatory events that were not detectable at the gene level, including stage-biased isoform usage and isoform switching. These events remodel protein domain architecture, and in some cases, alter predicted tertiary structure, suggesting functional divergence at the isoform level. Our findings uncover regulatory complexity that would be masked by gene-level analyses alone and highlight the importance of characterizing isoform dynamics when investigating insect development. This work provides a foundation for future studies aimed at isoform-level regulation and provides a resource for exploring isoform-specific targets in molecular pest management strategies.

## Supplementary Material

Supplemental Material

Supplemental Material

## Data Availability

These sequence data have been submitted to the NCBI SRA database under BioProject number PRJNA1037564.
