# Supplementary material for: Isoform-resolved transcriptomics reveals stage-specific isoform switching and protein domain remodeling in Helicoverpa armigera
Source: Transcription. 2026 Jul 27;17(2):163–80. doi: 10.1080/21541264.2026.2708591 (PMC13502018; doi:10.1080/21541264.2026.2708591)
Supplement: Supplementary file 1 [file KTRN_A_2708591_SM5949.docx]

 **Supplementary Figures**

B

A

**Supplementary Figure 1.** (A) Dot plot illustrates the enriched KEGG pathways among genes with isoform switching. Dot size represents the number of genes associated with each pathway, while dot color indicates the adjusted p-value significance level. (B) Bar plot illustrates the enrichment of GO terms associated with isoform switched genes. Functional terms were grouped and color coded into three categories: biological process (BP), molecular function (MF), and cellular components (CC).
